# The efficacy and safety of combined methotrexate with anti-vascular endothelial growth factor therapy in treatment of diabetic macular edema: A protocol for systematic review and meta-analysis

**DOI:** 10.1097/MD.0000000000025684

**Published:** 2021-05-14

**Authors:** Yuzhi Bao, Xiaolei Lu, Yuwei Zhou

**Affiliations:** a Department of Ophthalmology, Enshi Huiyi Eye Hospital; b Department of Laboratory, Enshi Huiyi Medical Laboratory, Hubei, China.

**Keywords:** anti-vascular endothelial growth factor, diabetic macular edema, meta-analysis, methotrexate, protocol, systematic review

## Abstract

**Objective:**

There is no review or meta-analysis to compare the efficacy and safety of methotrexate plus anti-vascular endothelial growth factor (anti-VEGF) therapy in patients with diabetic macular edema (DME). It is worthy to critically review the evidence of the assessment of combined therapies to inform clinical practice. Therefore, the purpose of this study was to compare the efficacy and safety of methotrexate plus anti-VEGF therapy in the treatment of DME and to provide evidence for clinical practice.

**Methods:**

The electronic databases of EMBASE, PubMed, Cochrane Library, and Web of Science were searched from the inception to April 2021 using the following key terms: “diabetic macular edema,” “methotrexate,” and “anti-vascular endothelial growth factor,” for all relevant studies. Additionally, the reference lists from published original articles and relevant reviews were assessed to identify more relevant studies. Only English publications were included. Data were extracted by review of each study for population, mean age, gender, follow-up duration, study design, publishing date, characteristics, and outcomes assessment. The present study was performed using Review Manager (RevMan Version 5.3, The Cochrane Collaboration, Copenhagen, Denmark).

**Results:**

We hypothesized that combined therapies would provide better therapeutic benefits compared to single method.

**OSF registration number:**

10.17605/OSF.IO/APD6 V.

## 1 Introduction

Diabetic macular edema (DME), a specific form of diabetic retinopathy, is a highly specific microvascular complication of diabetes in the eye. DME is a thickening of the retina in 2 diameters of the optic nerve starting at the center of the macula, functionally the most important part of the eye.^[1]^ This is due to the breakdown of the blood-retinal barrier, abnormal microvascular permeability and subsequent infiltration of fluid and plasma components into the macular layer. The structural changes of DME are characterized by the accumulation of fluid and hard exudates in the outer clump and inner core layers of macula, and the formation of fluid-filled cystic spaces.^[2,3]^

A large number of randomized clinical trials have identified intravitreal anti-vascular endothelial growth factor (anti-VEGF) therapy as a first-line treatment for visual impairment in DME.^[4,5]^ Although anti-VEGF injections have a positive effect on vision and retinal thickening, DME can persist in some eyes.^[6]^ Methotrexate is an anti-tumor and anti-inflammatory drug that has been used to treat various malignant tumors and rheumatic diseases. In ophthalmology, systemic and intraocular methotrexate has been successfully used for nodular uveitis, uncertain uveitis, non-Hodgkin's lymphoma, and primary central nervous system lymphoma.^[7]^ Recently, several trials have used intravitreal methotrexate in the treatment of persistent DME and have shown that intravitreal methotrexate injection has anti-inflammatory effects and is significantly effective in the treatment of persistent DME.^[8,9]^

Nowadays, there is no review or meta-analysis to compare the efficacy and safety of methotrexate plus anti-VEGF therapy in patients with DME. It is worthy to critically review the evidence of the assessment of combined therapies to inform clinical practice. Therefore, the purpose of this study was to compare the efficacy and safety of methotrexate plus anti-VEGF therapy in the treatment of DME and to provide evidence for clinical practice. We hypothesized that combined therapies would provide better therapeutic benefits compared to single method.

## 2 Materials and methods

### 2.1 Selection of studies

The systematic review protocol has been registered on Open Science Framework registries. Two independent investigators followed The Preferred Reporting Items for Systematic Reviews and Meta-Analyses reporting guidelines and the recommendations of the Cochrane Collaboration to conduct this meta-analysis. The electronic databases of EMBASE, PubMed, Cochrane Library, and Web of Science were searched from the inception to April 2021 using the following key terms: “diabetic macular edema,” “methotrexate,” and “anti-vascular endothelial growth factor,” for all relevant studies. Additionally, the reference lists from published original articles and relevant reviews were assessed to identify more relevant studies. Only English publications were included. Ethical approval was not necessary because the present meta-analysis was performed on the basis of previous published studies.

### 2.2 Inclusion and exclusion criteria

Study included in this systematic review and meta-analysis had to meet all of the following inclusion criteria in the PICOS order:

1. Participants: DME patients;
2. Intervention: patients received methotrexate plus anti-VEGF therapy;
3. Comparator: patients received methotrexate or anti-VEGF therapy;
4. Outcomes: outcomes which assessed changes in the best corrected visual acuity logMAR, changes in the central subfield thickness, maximum retinal thickness, and central macular volume;
5. Study design: randomized controlled trials (RCTs).

The exclusion criteria were as follows:

1. studies which did not assessed the above outcomes;
2. no direct comparison of combined therapies and single method;
3. studies with the following types: case reports, comments or letters, biochemical trials, protocols, conference abstracts, reviews, and retrospective studies or prospective non-randomized studies.

### 2.3 Data extraction

Data were extracted by review of each study for population, mean age, gender, follow-up duration, study design, publishing date, characteristics, and outcomes assessment. The 2 reviewers created a study-specific speadsheet in Excel (Microsoft Corp., USA) for data collection. Data extraction was performed independently, and any conflict was resolved before final analysis. Any disagreements between the 2 reviewers were discussed and, if necessary, the third author was referred to for arbitration. If the data were missing or could not be extracted directly, authors were contacted by email. Otherwise, we calculated them with the guideline of Cochrane Handbook for Systematic Reviews of Interventions 5.1.0. If necessary, we would abandon the extraction of incomplete data.

### 2.4 Risk of bias assessment

Two independent reviewers evaluated the risk of bias of the included RCTs on the basis of the guidelines of the Cochrane Handbook for Systematic Reviews of Interventions 5.1.0 by using Cochrane Collaboration's tool for assessing the risk of bias. The score consisted of 7 items, including random sequence generation, allocation concealment, blinding of participants and personnel, blinding of outcome assessment, incomplete outcome data, selective reporting, and other bias.

### 2.5 Quality of evidence

We assessed the quality of evidence of the outcomes by using the Grading of Recommendations Assessment, Development, and Evaluation (GRADE) system that included the following items: risk of bias, stable effect, inconsistency, and imprecision. The recommended levels of evidence were classified into 4 categories, including very low, low, moderate, and high. Any disagreement was resolved through discussion with a third reviewer.

### 2.6 Data synthesis

The present study was performed using Review Manager (RevMan Version 5.3, The Cochrane Collaboration, Copenhagen, Denmark). Risk ratios with a 95% confidence interval or standard mean difference with 95% CI were assessed for dichotomous outcomes or continuous outcomes, respectively. *P* < .05 was set as the level of significance. A result was also considered statistically significant if “1” was not included in the 95% CI of risk ratios or “0” was not included in the 95% confidence interval of standard mean difference. The *Q* test and *I*^2^ statistic were used to assess heterogeneity. A fixed-effects model was used if *P* > .1 and *I*^2^ < 50%, which indicated homogeneity. On the contrary, a random-effects model was used when *P* ≤ .1 or *I*^2^ ≥ 50%. The origins of heterogeneity were investigated using the sensitivity analysis.

## 3 Discussion

Anti-VEGF injections are typically standard treatment for eyes with DME and vision impairment. Nowadays, there is no review or meta-analysis to compare the efficacy and safety of methotrexate plus anti-VEGF therapy in patients with DME. It is worthy to critically review the evidence of the assessment of combined therapies to inform clinical practice. Therefore, the purpose of this study was to compare the efficacy and safety of methotrexate plus anti-VEGF therapy in the treatment of DME and to provide evidence for clinical practice. We hypothesized that combined therapies would provide better therapeutic benefits compared to single method.

## Author contributions

**Conceptualization:** Xiaolei Lu.

**Data curation:** Yuzhi Bao, Xiaolei Lu.

**Formal analysis:** Yuzhi Bao, Xiaolei Lu.

**Funding acquisition:** Yuwei Zhou.

**Investigation:** Yuzhi Bao, Xiaolei Lu.

**Methodology:** Xiaolei Lu.

**Project administration:** Yuwei Zhou.

**Resources:** Yuwei Zhou.

**Software:** Yuzhi Bao, Xiaolei Lu.

**Supervision:** Yuwei Zhou.

**Validation:** Yuzhi Bao.

**Visualization:** Xiaolei Lu.

**Writing – original draft:** Yuzhi Bao.

**Writing – review & editing:** Yuwei Zhou.
